# Reduced-dose dexamethasone premedication for weekly paclitaxel: a retrospective cohort study of early hypersensitivity reactions and steroid-related toxicity

**DOI:** 10.1007/s00520-026-10933-2

**Published:** 2026-06-29

**Authors:** Jeayoon Lee, Yijin An, In-Wha Kim, Minoh Ko, Jung Mi Oh

**Affiliations:** 1 College of Pharmacy and Research Institute of Pharmaceutical Sciences, Seoul National University, 1 Gwanak-Ro, Gwanak-Gu, Seoul, 08826 Republic of Korea; 2 College of Pharmacy, Daegu Catholic University, Gyeongsan-Si, 38430 Gyeongbuk Republic of Korea; 3 College of Pharmacy, Natural Products Research Institute, Seoul National University, Seoul, Republic of Korea

**Keywords:** Taxane, Premedication, Dose reduction, Hypersensitivity, Adverse events

## Abstract

**Purpose:**

Taxane-induced hypersensitivity reactions (HSRs) are highly prevalent, necessitating premedication with dexamethasone; however, prolonged or high-dose exposure to dexamethasone increases the risk of adverse effects. This study evaluated whether a reduced-dose dexamethasone regimen effectively prevents HSRs while minimizing steroid-related toxicity during weekly paclitaxel chemotherapy.

**Methods:**

This retrospective cohort study utilized electronic health records to evaluate patients receiving weekly paclitaxel. We compared a low-dose (LD) dexamethasone regimen (10 mg at the first infusion, followed by 4 mg weekly) with a conventional high-dose (HD) regimen (10 mg weekly). The primary outcome was the incidence of HSRs during the second paclitaxel infusion, analyzed using non-inferiority testing with a predefined margin of 2%. Propensity score-based inverse probability of treatment weighting was employed to control confounding, with missing data addressed via multiple imputation. Secondary outcomes included the incidence of steroid-related adverse events (AEs) such as hyperglycemia, insomnia, and serious bacterial infections, evaluated using Cox proportional hazards models adjusted for relevant clinical factors.

**Results:**

The LD regimen was non-inferior to the HD regimen for preventing HSRs (weighted incidence, 0.72% vs. 1.18%; risk difference −0.46%; 95% CI −1.26% to 0.53%). The HD group had a significantly higher risk of hyperglycemia (adjusted HR [aHR] 1.54, 95% confidence interval [CI] 1.15 to 2.07). While insomnia (aHR 1.53, 95% CI 0.93 to 2.51) and serious bacterial infections (aHR 1.19, 95% CI 0.99 to 1.44) occurred more frequently in the HD group, these trends did not reach statistical significance.

**Conclusion:**

A low-dose dexamethasone premedication regimen was non-inferior to the conventional regimen in preventing HSRs and was associated with a lower risk of corticosteroid-associated hyperglycemia.

**Supplementary Information:**

The online version contains supplementary material available at 10.1007/s00520-026-10933-2.

## Introduction

Taxanes, including paclitaxel and docetaxel, are widely used to treat solid tumors. In particular, weekly paclitaxel regimens have become a widely used treatment approach, particularly in breast cancer, owing to their favorable balance between efficacy and tolerability [1]. However, taxanes frequently cause hypersensitivity reactions (HSRs), which range from mild cutaneous symptoms to potentially life-threatening anaphylaxis and death in rare cases [2–4]. The etiology of taxane-induced HSRs is multifactorial, involving both direct and indirect immune mechanisms such as complement activation by solvents such as Cremophor EL and polysorbate 80, histamine release, and possible IgE- or IgG-mediated responses [5, 6]. Notably, taxane-induced HSRs typically occur during the initial cycles of treatment, most commonly during the first or second infusion, with a substantially lower risk in subsequent administrations following an uneventful exposure [5].

To mitigate the risk of HSRs, standard premedication protocols involving dexamethasone and antihistamines are commonly employed [7–9]. In addition to its established role in preventing HSRs, dexamethasone is also administered as premedication to reduce the risk of other adverse events (AEs), such as fluid retention and peripheral edema, which primarily result from increased capillary permeability [10].

Although dexamethasone effectively suppresses inflammatory responses, stabilizes mast cells, and reduces capillary permeability [8], repeated or high-dose administration can lead to AEs, including insomnia, mood disturbances, hyperglycemia, and infection, which are of particular concern in patients with cancer who have multiple comorbidities [11].

The prescribing information for paclitaxel recommends administering 20 mg of oral dexamethasone at 12 h and 6 h prior to infusion [12]. However, dexamethasone dose reduction is commonly adopted in clinical practice, particularly in weekly regimens, to minimize steroid-related toxicities [13, 14]. Observational studies have demonstrated the feasibility of reducing or omitting dexamethasone premedication after initial HSR-free cycles in biweekly, triweekly, and mixed regimens [15–19]. Weekly regimens, however, involve prolonged steroid exposure and are associated with a higher risk of complications such as severe infections [20, 21]. Nevertheless, previous studies of weekly protocols were limited by small cohorts (fewer than 100 patients), reducing statistical power to detect rare AEs and limiting the external validity of their findings [22, 23].

To address concerns regarding cumulative steroid exposure, Seoul National University Hospital (SNUH) revised its weekly paclitaxel premedication protocol in 2019. Previously, patients received 10 mg of dexamethasone before every paclitaxel infusion. Under the revised protocol, 10 mg is administered only before the first dose, and 4 mg before subsequent infusions. Leveraging this institutional change and the limited evidence available for weekly regimens, we hypothesized that the low-dose (LD) dexamethasone regimen would be non-inferior to the high-dose (HD) regimen for preventing HSRs, while offering an improved safety profile with fewer steroid-related AEs.

## Methods

### Study design and population

This retrospective cohort study at SNUH was conducted in accordance with the Strengthening the Reporting of Observational Studies in Epidemiology (STROBE) guidelines.

Eligible patients were those who received at least two weekly paclitaxel infusions between January 1, 2014, and May 31, 2018, or between January 1, 2020, and May 31, 2024, with infusion intervals ranging from 5 to 13 days. Sample size adequacy was determined a priori using a non-inferiority margin of 2%, a one-sided α of 0.05, and 80% statistical power. This calculation indicated that a minimum of 475 patients per group was required.

Premedication was administered intravenously 30 min before each paclitaxel infusion. Patients in the HD group received dexamethasone 10 mg, famotidine 20 mg, and pheniramine 45.5 mg (or an equivalent antihistamine). In the LD group, patients received dexamethasone 10 mg before the first infusion and 4 mg before all subsequent infusions, with the same antihistamine and famotidine regimen as the HD group. Patients who experienced severe HSRs requiring desensitization were excluded from the analytic cohort. Detailed inclusion and exclusion criteria are presented in Supplementary Table S1.

### Data collection

Data were collected from the Seoul National University Hospital Patients Research Environment (SUPREME), which is an institutional common data warehouse of electronic medical records (EMRs).

Demographics, including sex, age, height, weight, body mass index (BMI), and body surface area (BSA), as well as chemotherapy-related factors, such as regimen, paclitaxel dose, and prior surgery or radiotherapy, were collected. Comorbidities were defined using laboratory values and International Classification of Diseases (ICD)−10 codes: diabetes mellitus by a combination of random glucose > 200 mg/dL and HbA1c ≥ 6.5%, or ICD-10 codes E11–E14; hypertension by ICD-10 code I10; and baseline insomnia by prior prescriptions of insomnia medications. HSR-related factors, including documented respiratory/allergy-related history (ICD-10 codes J44–J46, L20) and baseline complete blood count (CBC) values, were also collected.

Dexamethasone exposure was assessed using drug name, daily dose, administration date, and administration status. HSRs were identified by reviewing nursing and outpatient records for patient-reported symptoms. AEs were captured using operational criteria based on laboratory values, medication use; diagnostic codes and microbiological test results were used where applicable. Detailed variable definitions and coding criteria are provided in Supplementary Table S2.

### Outcome

The primary outcome was the incidence of HSRs during the second infusion, as evaluated according to the National Cancer Institute’s Common Terminology Criteria for Adverse Events (CTCAE) version 5.0 [24]. An HSR was defined as a reaction temporally associated with paclitaxel administration, presenting with at least two characteristic symptoms (e.g., urticaria, bronchospasm, or hypotension), and not attributable to alternative causes. Detailed symptom descriptions and grading criteria are provided in Supplementary Table S3.

Secondary outcomes included dexamethasone-related AEs, specifically hyperglycemia, insomnia, and serious bacterial infections, observed within 30 days of the final paclitaxel dose (Supplementary Table S4).

### Statistical analysis

Categorical variables, including sex, comorbidities, previous chemotherapy, cancer type, cancer stage, and chemotherapy regimen, were analyzed using the Chi-square test. Continuous variables, such as age, BSA, BMI, CBC parameters, and dexamethasone exposure, were assessed for normality and homogeneity of variance using the Shapiro–Wilk test and Levene’s test, respectively; subsequent comparisons were performed using independent-samples t-tests or Mann–Whitney U tests, as appropriate.

#### Primary outcome analysis

The incidence of HSRs of any grade was reported for the first infusion as a baseline comparison. For the primary outcome, a non-inferiority analysis was conducted to evaluate whether the LD dexamethasone regimen was non-inferior to the HD regimen regarding second-infusion HSR prevention, using a non-inferiority margin of 2%. The non-inferiority margin was pre-specified at 2.0% based on clinical consensus and prior studies of taxane-induced HSRs, where absolute differences in this range have been used to evaluate the safety of premedication tapering [21, 25]. This margin was considered clinically acceptable given the potential benefit of reducing cumulative steroid toxicity. Crude risk differences (RDs) and corresponding 95% confidence intervals (CIs) were calculated for the second-infusion incidence. To control for potential confounding, covariates were prespecified based on clinical expertise using a directed acyclic graph (DAG) (Supplementary Fig. S1A). Propensity scores were estimated via logistic regression, incorporating age, BMI, absolute neutrophil count (ANC), lymphocyte count, prior radiotherapy or chemotherapy, allergy-related history, cancer stage and type, chemotherapy regimen, and paclitaxel dose.

Subsequently, stabilized inverse probability of treatment weighting (IPTW) was applied, with weights truncated at the 1st and 99th percentiles to minimize the influence of extreme values. Covariate balance was assessed using standardized mean differences (SMD), with values < 0.1 indicating adequate balance. Model discrimination was evaluated by the area under the receiver operating characteristic curve (AUC), with values > 0.7 considered acceptable.

#### Secondary outcome analysis

The association between dexamethasone dosage and the risk of steroid-related AEs was evaluated using univariable and multivariable Cox proportional hazards (PH) models. Covariates were pre-specified for each outcome based on DAGs (Supplementary Figs. S1B–S1D) and included any imbalanced baseline variables. The PH assumption was verified using Schoenfeld residuals; in cases of violation, stratified Cox models or models incorporating interaction terms with log-transformed time were applied, as appropriate.

As an exploratory analysis, the incidence of *Pneumocystis jirovecii* pneumonia (PJP), a rare but potentially fatal opportunistic infection associated with steroid use, was compared between groups using the chi-square test (Supplementary Table S4).

#### Sensitivity analyses

To evaluate the robustness of our primary findings, several sensitivity analyses were performed. First, nonparametric bootstrap resampling with 1,000 iterations was conducted on the IPTW-adjusted cohort to ensure the stability of the estimated RDs. Second, to assess the potential impact of missing data, all analyses were repeated using complete-case datasets and multiple imputation (*m* = 50) as alternative approaches. Finally, the primary outcome was redefined as Grade ≥ 3 events (per CTCAE v5.0) to evaluate non-inferiority under a stricter definition, focusing on severe or life-threatening reactions.

## Results

### Study population

**Fig. 1:**
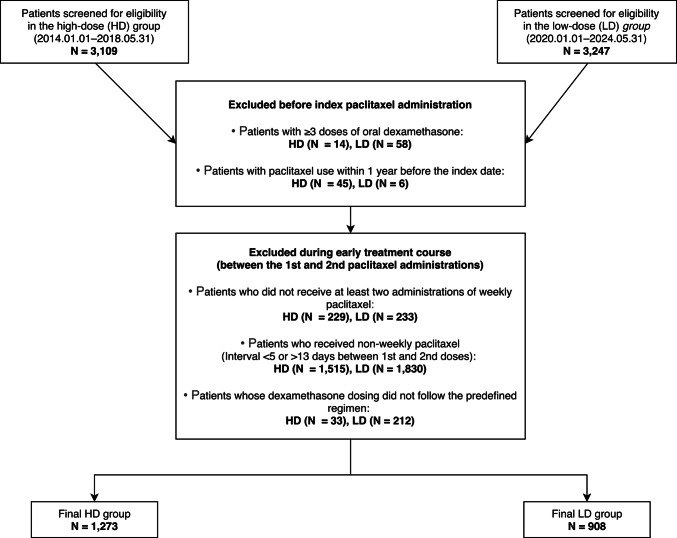
Study flow diagram. Flowchart depicting patient selection and assignment to the high-dose (HD) and low-dose (LD) dexamethasone premedication groups. Patients were screened during two distinct periods: HD group (2014.01.01–2018.05.31) and LD group (2020.01.01–2024.05.31), with a 1-year washout interval between cohorts. Eligible patients received at least two doses of paclitaxel, administered on a weekly schedule (defined as a 5–13 day interval between the first and second doses). The HD group received 10 mg dexamethasone prior to each infusion, while the LD group received 10 mg at the first infusion and 4 mg at the second. After applying exclusion criteria, the final analytic cohorts included 1,273 patients in the HD group and 908 patients in the LD group

A total of 2,181 patients met the eligibility criteria and were included in the analysis, with 1,273 patients assigned to the HD dexamethasone group and 908 to the LD group. The patient selection process and baseline characteristics are summarized in Fig. 1 and Table 1, respectively. As expected, the HD group received a substantially higher cumulative dexamethasone dose (93.6 vs 41.3 mg; *p* < 0.001) over a longer treatment duration (75.4 vs 69.5 days; *p* = 0.007). To ensure comparability between the groups, IPTW was employed, achieving an adequate covariate balance for all analyzed factors (SMD < 0.1) (Supplementary Figure S2).

**Table 1:** Patient characteristics

	HD (*N* = 1,273)	LD (*N* = 908)	*p*-value
Age**,** mean (SD), years	59.52 (11.50)	61.47 (11.65)	** < 0.001**
Sex, *n* (%)			0.005
Male	636 (49.96%)	510 (56.17%)	
Female	637 (50.04%)	398 (43.83%)	
Height, mean (SD), cm	161.4 (8.2)	162.4 (8.0)	0.005
Weight, mean, (SD), kg	56.7 (10.4)	57.9 (10.8)	0.011
Body surface area, mean, (SD), m^2^	1.62 (0.16)	1.64 (0.17)	0.272
Body mass index, mean, (SD), kg/m^2^	21.80 (3.78)	21.95 (3.74)	0.278
White blood count, median, (IQR), %	6.26 (4.74–8.17)^a^	6.12 (4.8–7.98)^b^	0.291
Absolute neutrophil count, median, (IQR), × 10^6^/L	3,853 (2,745–5,435.5)^c^	3,859 (2,668–5,424)^d^	0.684
Lymphocyte count, median, (IQR), %	22.1 (15.2–30)^a^	22 (15.1–30.4)^b^	0.895
Hemoglobin, median, (IQR), %	11.5 (10.5–12.6)^c^	11.6 (10.35–12.6)^d^	0.498
Comorbidities, *n* (%)			
Diabetes	143 (11.23%)	113 (12.44%)	0.424
Hypertension	98 (7.70%)	82 (9.03%)	0.300
Insomnia	119 (9.35%)	57 (6.28%)	0.012
Asthma/COPD/allergy	2 (0.16%)	51 (5.62%)	** < 0.001**
Previous chemotherapy, *n* (%)	971 (76.28%)	624 (68.72%)	** < 0.001**
Cancer type, *n* (%)			0.076
Lung	405 (31.81%)	309 (34.03%)	
Breast	387 (30.40%)	230 (25.33%)	
Gastrointestinal	367 (28.83%)	267 (29.41%)	
Esophagus	92 (7.23%)	83 (9.14%)	
Others^e^	22 (1.73%)	19 (2.09%)	
Cancer stage, *n* (%)			** < 0.001**
Stage 1–3	565 (44.38%)	540 (59.47%)	
Stage 4	708 (55.62%)	368 (40.53%)	
Chemotherapy regimen, *n* (%)			** < 0.001**
Weekly paclitaxel only	814 (63.94%)	254 (27.97%)	
Weekly paclitaxel + carboplatin	360 (28.28%)	388 (42.73%)	
Weekly paclitaxel + ramucirumab	62 (4.87%)	236 (25.99%)	
Weekly paclitaxel + trastuzumab	10 (0.79%)	25 (2.75%)	
Weekly paclitaxel + triweekly trastuzumab	25 (1.96%)	0 (0.00%)	
Others^f^	2 (0.16%)	4 (0.44%)	
Dexamethasone cumulative dose, mean, (SD), mg	93.6 (73.9)	41.3 (39.2)	** < 0.001**
Dexamethasone duration, mean, (SD), day	75.4 (74.5)	69.5 (89.0)	0.007
Paclitaxel dose, mean, (SD), mg	108.6 (22.4)	104.2 (24.4)	** < 0.001**
Number of paclitaxel infusions, median, (IQR)	6 (5–12)	6 (5–12)	0.061
HSRs at the first infusion, *n* (%)	22 (1.73)	28 (3.08)	0.052

*HD*, high-dose group; *LD*, low-dose group

^a^Missing data for 435 participants (34.2%)

^b^Missing data for 435 participants (47.9%)

^c^Missing data for 440 participants (34.6%)

^d^Missing data for 437 participants (48.1%)

^e^Urinary, angiosarcoma, ovarian, Kaposi’s sarcoma, adenocarcinoma, neuroendocrine, extramammary Paget’s disease, unknown

^f^Paclitaxel with avelumab, paclitaxel with cisplatin, paclitaxel with triweekly carboplatin

Bold values indicate *p* 0.001

### Primary outcome

Table 2 summarizes the HSR frequencies and non-inferiority results. On the first infusion, when both groups received 10 mg of dexamethasone, HSRs occurred in 22 patients (1.73%) in the HD group and 28 patients (3.08%) in the LD group (*p* = 0.052). Of the 22 patients in the HD group with a first-infusion HSR, all continued the 10 mg regimen; among them, 4 patients (18.2%) experienced a recurrent HSR at the second infusion, while 11 patients developed a new-onset HSR only at the second infusion. In the LD group, all 28 patients with a first-infusion HSR proceeded with the reduced dose (4 mg) as per protocol. Despite the dose reduction, only 3 of these patients (10.7%) experienced a recurrent HSR at the second infusion, while 4 patients developed a new-onset HSR. Therefore, on the second infusion, HSRs were reported in 15 patients (1.18%) in the HD group and 7 patients (0.77%) in the LD group (*p* = 0.471). Detailed individual clinical characteristics and management of these second-infusion HSR cases are presented in Table 3.

**Table 2:** Primary and sensitivity analyses for hypersensitivity reactions

Outcome	HD (*N* = 1,273)	LD (*N* = 908)	Risk difference (LD − HD)	95% CI	Non-inferiority^a^
HSRs at the second infusion, *n* (%)	15 (1.18)	7 (0.77)	−0.41	−1.26 to 0.53	Achieved
HSRs at the second infusion (IPTW-weighted), %	1.18	0.72	−0.46	−1.35 to 0.43	Achieved
HSRs at the second infusion (bootstrap, *N* = 1,000), %	1.15	0.68	−0.47	−1.19 to 0.35	Achieved
HSRs at the second infusion (complete-case analysis), %	1.06^†^	0.84^‡^	−0.22	−1.34 to 0.89	Achieved
HSRs at the second infusion (multiple imputation, m = 50), %	1.18	0.75	−0.43	−1.24 to 0.49	Achieved
Grade ≥ 3 HSRs at the second infusion, *n* (%)	5 (0.39)	4 (0.44)	0.05	−0.54 to 0.77	Achieved

*HD*, high-dose group; *LD*, low-dose group; *HSR*, hypersensitivity reaction; *IPTW*, inverse probability of treatment weighting; *CI*, confidence interval

^a^Non-inferiority was assessed using a prespecified margin of 2% (risk difference)

^†^Complete-case analysis was performed on 833 patients after excluding those with missing data

^‡^Complete-case analysis was performed on 471 patients after excluding those with missing data

**Table 3:** Clinical characteristics and management of day 2 hypersensitivity reactions

Group	Patient no	Sex/age	Primary cancer	Regimen	Paclitaxel dose (mg/m^2^)	HSR at first infusion	Grade of HSR at second infusion^a^	Key symptoms	Intervention^b^
HD	1	F/48	Breast	Weekly Paclitaxel	80	No	2	Rash	Oral symptomatic treatment after infusion
HD	2	F/63	Breast	Weekly Paclitaxel	80	Yes	1	Sweating, Hypotension	No intervention
HD	3	F/39	Breast	Weekly Paclitaxel	80	No	2	Rash	Oral symptomatic treatment after infusion
HD	4	M/53	Lung	Weekly Paclitaxel	80	No	3	General discomfort	Chemotherapy interrupted, IV antihistamine/steroid administered, Chemotherapy resumed
HD	5	M/43	GI	Weekly Paclitaxel	80	Yes	2	Chest tightness, Back pain	Chemotherapy infusion rate adjustment
HD	6	M/68	GI	Weekly Paclitaxel	64	No	1	Dyspepsia, Rash	No intervention
HD	7	F/43	Breast	Weekly Paclitaxel	80	Yes	1	Chest tightness	No intervention
HD	8	F/39	Breast	Weekly Paclitaxel	80	No	2	Urticaria, Pruritus	Oral symptomatic treatment after infusion
HD	9	F/56	Breast	Weekly Paclitaxel	80	No	2	Rash	Oral symptomatic treatment after infusion
HD	10	F/57	GI	Weekly Paclitaxel	80	No	3	Flushing, Chest tightness	Chemotherapy interrupted, IV antihistamine/steroid administered, Chemotherapy resumed
HD	11	M/74	Esophagus	Weekly Paclitaxel + Carboplatin	50	No	3	Pain, Chills, Rash, Back pain	Chemotherapy interrupted, IV antihistamine/steroid administered, Chemotherapy resumed
HD	12	M/60	Lung	Weekly Paclitaxel + Carboplatin	50	No	2	Pruritus, Rash	Oral symptomatic treatment after infusion
HD	13	F/33	Breast	Weekly Paclitaxel + Herceptin	80	Yes	2	Urticaria	Oral symptomatic treatment after infusion
HD	14	M/58	Lung	Weekly Paclitaxel + Carboplatin	50	No	3	Chest discomfort, Dyspnea	Chemotherapy interrupted, IV antihistamine/steroid administered, Chemotherapy resumed
HD	15	M/51	Lung	Weekly Paclitaxel	80	No	3	Dyspnea, Chest tightness	Chemotherapy interrupted, IV antihistamine/steroid administered, Chemotherapy resumed
LD	1	F/39	Breast	Weekly Paclitaxel	80	Yes	3	Urticaria	Chemotherapy interrupted, IV antihistamine/steroid administered, Chemotherapy resumed
LD	2	M/77	Lung	Weekly Paclitaxel + Carboplatin	35	No	3	Flushing	Chemotherapy interrupted, IV antihistamine/steroid administered, Chemotherapy resumed
LD	3	F/28	Breast	Weekly Paclitaxel	80	Yes	2	Dizziness, Sweating	Chemotherapy interrupted, Chemotherapy resumed
LD	4	M/59	Lung	Weekly Paclitaxel + Carboplatin	50	No	3	General discomfort	Chemotherapy interrupted, IV antihistamine/steroid administered, Chemotherapy resumed
LD	5	F/54	Breast	Weekly Paclitaxel	80	No	2	Rash	Oral symptomatic treatment after infusion
LD	6	M/71	Lung	Weekly Paclitaxel + Carboplatin	50	No	3	Flushing, Rash, Pruritus	Chemotherapy interrupted, IV antihistamine/steroid administered, Chemotherapy resumed
LD	7	F/52	Lung	Weekly Paclitaxel + Carboplatin	50	Yes	2	Urticaria	Oral symptomatic treatment after infusion

*F*, female; *GI*, gastrointestinal; *HD*, high-dose dexamethasone; *HSR*, hypersensitivity reaction; *IV*, intravenous; *LD*, low-dose dexamethasone; *M*, male

^a^Grading was based on the Common Terminology Criteria for Adverse Events (CTCAE) version 5.0

^b^Management includes pharmacological interventions such as Avil (pheniramine) and Cortisolu (hydrocortisone)

The crude RD (LD − HD) for second-infusion HSRs was − 0.41% (95% CI, − 1.26 to 0.53), with the upper bound of the confidence interval falling well below the pre-specified non-inferiority margin of 2%. After IPTW, the weighted incidence of second-infusion HSRs was 1.18% in the HD group and 0.72% in the LD group (RD, − 0.46%; 95% CI, − 1.35 to 0.43), confirming the criteria for non-inferiority. Covariate balance before and after weighting is shown in Supplementary Fig. S2, with all covariates achieving an SMD < 0.1 after weighting. The propensity score model demonstrated good discrimination, with an AUC of 0.77 (Supplementary Fig. S3).

### Secondary outcomes

**Fig. 2:**
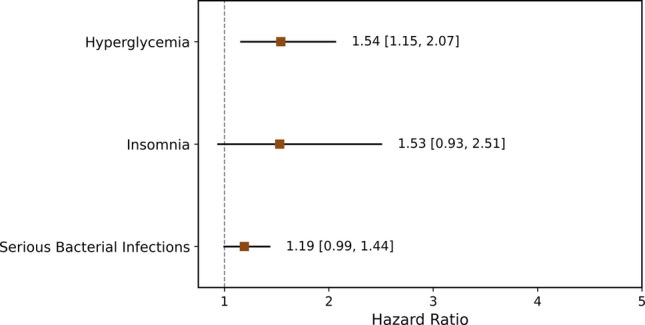
Adjusted hazard ratios (HRs) for dexamethasone-related adverse events (AEs). Multivariable Cox proportional hazards models were used to estimate adjusted hazard ratios (HRs) and 95% confidence intervals (CIs) for (a) hyperglycemia, (b) insomnia, and (c) serious bacterial infections. Relevant covariates were included in each model, with stratification or time-varying terms applied when the proportional hazards assumption was violated. HRs are plotted on the x-axis; squares indicate point estimates and horizontal lines represent 95% confidence intervals. Numeric labels represent the HRs and their 95% CIs

Table 4 summarizes the risk of AEs according to the dexamethasone dose group. For the hyperglycemia analysis, patients without available glucose measurements were excluded, resulting in 1,261 patients in the HD group and 901 in the LD group. The incidence of hyperglycemia was higher in the HD group (10.15%) than in the LD group (7.66%). After adjusting for age, sex, BMI, and diabetes history, the HD group had a significantly increased risk of hyperglycemia (adjusted hazard ratio [aHR] 1.54, 95% CI 1.15 to 2.07; Fig. 2).

**Table 4:** Risk of adverse events according to the dexamethasone dose group (reference group: LD)

Outcome	Events (HD), *n* (%)	Events (LD), *n* (%)	Crude HR (95% CI)	Adjusted HR (95% CI)
Hyperglycemia^a^	128 (10.15)^†^	69 (7.66)^†^	1.33 (0.99–1.78)	1.54 (1.15–2.07)
Insomnia^b^	59 (4.63)	23 (2.53)	1.81 (1.11–2.96)	1.53 (0.93–2.51)
Serious bacterial infections^c^	243 (19.09)	131 (14.43)	1.21 (1.00–1.45)	1.19 (0.99–1.44)

*CI*, confidence interval; *LD*, low dose; *HD*, high dose; *HR*, hazard ratio

^a^Cox proportional hazards model adjusted for age, sex, BMI, and diabetes history

^b^Stratified Cox model adjusted for age and sex and stratified by baseline insomnia history

^c^Cox model adjusted for age and absolute neutrophil count (ANC), which was collected each time during paclitaxel administration and modeled as a time-varying covariate. The model was stratified by cancer stage, and included an interaction term between the treatment group and log(time). Missing ANC data (28.7% in HD; 37.0% in LD) were imputed using an iterative regression-based method (scikit-learn’s Iterative Imputer). More than 99% of patients in both groups had at least two ANC measurements

^†^The number of patients for hyperglycemia differs because of the exclusion of patients without available glucose laboratory data (HD = 1,261; LD = 901)

Insomnia occurred in 4.63% and 2.53% in the HD and LD groups, respectively. Due to a violation of the PH assumption by baseline insomnia status, a stratified Cox model was applied. After adjusting for age, sex, and baseline insomnia history, the aHR for the HD group was 1.53 (95% CI, 0.93 to 2.51), which did not reach statistical significance (Fig. 2).

Serious bacterial infections occurred in 19.09% and 14.43% of patients in the HD and LD groups, respectively. To address PH assumption violations and the dynamic nature of risk, a stratified Cox model was employed, adjusting for age and ANC as a time-varying covariate, stratified by cancer stage, while incorporating a log-time interaction term. As shown in Fig. 2, the aHR at day 14—corresponding to the third paclitaxel administration and the first assessment point after the dexamethasone regimens diverged—was 1.19 (95% CI, 0.99 to 1.44). The HD group showed a higher risk of infection early in treatment (aHR 1.31 [95% CI, 1.08 to 1.60] at day 7; aHR 1.25 [95% CI, 1.03 to 1.51] at day 10), with this effect gradually decreasing over time (Supplementary Fig. S4).

In the exploratory analysis, the incidence of PJP was 1.34% (*n* = 17) in the HD group and 0.77% (*n* = 7) in the LD group, showing no statistically significant difference (*p* = 0.30; Table 4).

### Sensitivity analyses

Sensitivity analyses confirmed the robustness of the primary findings across various statistical assumptions and clinical definitions (Table 2). Nonparametric bootstrap resampling (1,000 iterations) in the IPTW-adjusted cohort yielded consistent results: weighted incidence rates were 1.15% for the HD group and 0.68% for the LD group, with an RD of − 0.47% (95% CI, − 1.19 to 0.35). When accounting for missing data, the complete-case analysis and multiple imputation yielded RDs of − 0.22% (95% CI, − 1.34 to 0.89) and − 0.43% (95% CI, − 1.24 to 0.49), respectively. Furthermore, when the outcome was restricted to Grade ≥ 3 HSRs, the RD was 0.05% (95% CI, − 0.54 to 0.77). In all analyses, the upper bounds of the 95% CIs remained below the pre-specified 2% non-inferiority margin.

## Discussion

This study evaluated the effectiveness and safety of an LD dexamethasone premedication regimen compared to the conventional HD regimen in patients receiving weekly paclitaxel. Previous studies have explored strategies to omit dexamethasone premedication in patients receiving taxane-based chemotherapy after initial uneventful cycles. A retrospective analysis of 449 breast cancer patients suggested that discontinuing dexamethasone after two uneventful cycles of paclitaxel was safe [18], and a prospective study in 125 patients on biweekly paclitaxel also reported that corticosteroid omission in later cycles did not increase HSRs if none occurred during earlier cycles [16]. However, the absence of a control cohort in these studies limited the robustness of their comparative conclusions.

Beyond the mere omission of steroids, several retrospective cohort studies have also investigated whether lower dexamethasone doses are sufficient for HSR prophylaxis. In a large EMR-based analysis of over 3,000 patients treated with paclitaxel or docetaxel, no significant association was observed between dexamethasone dose categories (0–10, < 10–20, or > 20 mg) and the incidence of HSRs [15]. Similarly, a study of gynecologic cancer patients found no outcome differences between dexamethasone doses above and below 10 mg [17]. These findings suggest that lower doses, particularly around 10 mg, may provide adequate prophylaxis for most patients.

Our study extends previous research by evaluating a specific step-down dexamethasone regimen, starting at 10 mg in the first infusion and tapering to 4 mg in subsequent infusions. We demonstrated that this LD strategy was non-inferior in preventing HSRs while providing an improved safety profile. This finding is particularly relevant for weekly paclitaxel regimens, where cumulative steroid exposure is inherently higher. Unlike earlier studies with heterogeneous treatment schedules, our results provide clear evidence supporting a gradual dose-reduction approach that balances efficacy and safety in real-world practice.

For the primary outcome, the non-inferiority analysis showed that the upper bound of the 95% CI for the RD was well within the pre-specified 2% margin. The robustness of these findings was supported by bootstrap resampling and consistent results from both complete-case analysis and multiple imputation, suggesting that the missingness in certain laboratory variables had a minimal impact on the estimated treatment effect. Regarding the 2% non-inferiority margin, while it might appear large relative to the observed 1% event rate, it was pre-specified based on clinical consensus and prior literature. Crucially, the upper bound of the 95% CI for the RD was only 0.43%, which is well below both the pre-specified margin and the observed event rate, suggesting that the findings are robust even under more conservative margin assumptions.

Notably, despite a higher prevalence of asthma, chronic obstructive pulmonary disease (COPD), or allergy history in the LD group (5.6% vs. 0.2%), adjusted analyses showed no increased risk of HSRs. However, the extreme scarcity of these conditions in the HD group presents a challenge of sparse data and limited overlap, which may render further statistical adjustment unstable or susceptible to over-interpretation. Nevertheless, as these conditions are established risk factors for hypersensitivity, this imbalance would be expected to bias the results toward a higher HSR risk in the LD group. In this context, the absence of an increased risk may suggest that the findings are not solely explained by this baseline difference. This is further supported by our granular analysis of first-infusion reactors; the recurrence rate in the LD group was numerically lower than in the HD group (10.7% vs. 18.2%), despite a 60% reduction in the dexamethasone dose reduction (Table 3). Such findings provide strong evidence that the 4 mg regimen offers sufficient prophylaxis even for patients who exhibited initial sensitivity. Nonetheless, residual confounding cannot be fully excluded.

Our focus on the second infusion as the primary endpoint is also clinically justified, as taxane-induced HSRs predominantly occur during the first two cycles [5, 26, 27]. Because immune tolerance often develops after these initial uneventful exposures, the risk of new-onset HSRs in later administrations is relatively low. Moreover, a longitudinal analysis of later cycles in a retrospective setting could be confounded by a ‘depletion of susceptibles’ effect and informative censoring, as patients sensitive to taxanes are progressively filtered out or undergo treatment modifications after early-phase reactions. Therefore, evaluating outcomes at the second infusion—the specific point where dexamethasone dosages first diverge—provides a clinically relevant assessment of the safety of the LD regimen. While late-onset HSRs were not formally analyzed, the robust safety observed at this critical divergence point, combined with the longitudinal reduction in cumulative steroid-related AEs, supports the overall feasibility of the LD strategy throughout the entire treatment course.

We also evaluated the safety profiles of the two groups by examining the incidence of steroid-related AEs, including hyperglycemia, insomnia, and serious bacterial infections. Considering that both the dose and duration of dexamethasone treatment can influence toxicity, we compared steroid exposure profiles between the two groups. While the HD group had a slightly longer treatment duration than the LD group (75.4 days vs. 69.5 days; *p* = 0.007), this difference was considered clinically negligible within the context of long-term weekly chemotherapy. This comparison allowed us to assess whether differences in dexamethasone dosage contributed to the incidence of AEs. A 30-day follow-up period after the final dose was established to adequately capture steroid-induced adverse reactions, including hyperglycemia, insomnia, and serious bacterial infections. This window was selected in consideration of weekly clinical visit patterns, the biological mechanisms of corticosteroid-related toxicity [28–30], and consistency with prior studies that used a 30-day adverse event monitoring period [31–33].

Hyperglycemia occurred more frequently in the HD group (aHR 1.54, 95% CI 1.15 to 2.07), after adjusting for age, sex, BMI, and history of diabetes [29, 34, 35]. This finding aligns with the dose-dependent metabolic effects of glucocorticoids [29, 34, 36], driven by insulin resistance and hepatic gluconeogenesis [36, 37]. While prior studies were often limited by small sample sizes or heterogeneous regimens [35, 38, 39], our study leveraged a large and homogeneous cohort to clarify this dose-dependent risk. These results support dexamethasone dose reduction as a key strategy to mitigate metabolic complications, which are known to adversely affect oncological outcomes [40].

Insomnia was also observed more frequently in the HD group, though the association did not reach statistical significance after adjustment (aHR 1.53, 95% CI 0.93 to 2.51). Interpretation of earlier studies comparing dexamethasone doses has been limited by a low number of reported events [39]. Given that disrupted sleep is associated with increased fatigue and reduced quality of life in cancer patients [41], minimizing steroid exposure may enhance overall patient well-being by reducing sleep disturbances [30].

Serious bacterial infections were more frequent in the HD group, with marginal statistical significance at day 14 (aHR 1.19, 95% CI 0.99 to 1.44). The association was stronger earlier in treatment and gradually attenuated over time, a pattern that may partly reflect censoring at the first infection event. The finding is biologically plausible given the known immunosuppressive effects of corticosteroids [42]. Given that chemotherapy-induced neutropenia is a major contributor to infection risk [43, 44], we incorporated ANC as a time-varying covariate to account for cycle-specific fluctuations while adjusting for age and cancer stage. We also defined serious bacterial infection as receipt of at least two intravenous antibiotic prescriptions within 7 days to reduce misclassification of empirical or precautionary treatment, consistent with prior studies [24, 35, 45–49]. Our findings align with prior research demonstrating reduced infection risk with lower-dose dexamethasone in weekly docetaxel regimens [38]. Notably, our analysis further strengthens the evidence by modeling ANC, a major determinant of infection risk, as a time-varying covariate [38, 42–44]. While our exploratory analysis of PJP did not reach statistical significance, the numerically higher incidence in the HD group (1.34% vs. 0.77%) is clinically noteworthy. Given the severe nature of such opportunistic infections in immunocompromised oncology patients, even a small reduction in steroid exposure could be a meaningful step toward enhancing patient safety. Taken together, these findings suggest that dexamethasone dose reduction may help lower the risk of infectious complications, which remain an important cause of morbidity in oncology.

Our findings support the feasibility of dexamethasone dose reduction in weekly paclitaxel regimens and align with a broader oncological trend toward minimizing corticosteroid exposure without compromising efficacy. A similar movement has been observed in pemetrexed-based therapy for metastatic non-small cell lung cancer, where a simplified single-dose dexamethasone regimen improved adherence compared to a traditional multiday schedule [50].

This study has several strengths. We leveraged a large, real-world EMR dataset with detailed laboratory values and chemotherapy regimens, enabling a focused analysis of the weekly paclitaxel schedule characterized by high cumulative steroid exposure. The substantial sample size provided sufficient statistical power for a robust non-inferiority assessment and the detection of clinically meaningful differences in steroid-related AEs. Furthermore, we employed outcome-specific analyses guided by DAGs to identify and adjust for relevant confounders, thereby strengthening the causal interpretability of our findings.

Despite its strengths, several limitations should be acknowledged. First, despite rigorous adjustments, the retrospective design may leave residual confounding and unmeasured or incompletely captured variables, such as undocumented allergy histories. Second, the relatively low incidence of HSRs resulted in wide CIs in certain subgroup analyses; nonetheless, the consistency of results across multiple analytical strategies supports the robustness of our findings. Third, as this was a non-concurrent historical cohort study with non-overlapping periods, temporal differences may have introduced systemic bias. Potential residual confounding may stem from changes in supportive care, advances in treatment, or modifications in EMR documentation over the 10-year period. However, this study was conducted at a tertiary referral hospital where chemotherapy administration and adverse event management generally follow standardized institutional protocols. Consequently, supportive care practices, apart from steroid dosage, remained largely consistent across the study periods. The LD group was treated during the COVID-19 pandemic, a period during which alterations in host immune function and infection patterns were frequently reported [51]. While taxane-induced HSRs are primarily immediate reactions mediated by complement activation and direct mast cell degranulation, which may be less dependent on adaptive immune modulation, the potential influence of pandemic-related factors cannot be fully excluded. To minimize temporal and measurement bias, we applied standardized HSR definitions based on CTCAE v5.0 and manually reviewed nursing and outpatient records across both periods to ensure consistency in outcome ascertainment. Regarding secondary outcomes, the infection analysis was restricted to serious bacterial infections to minimize confounding from broader shifts in viral infection patterns during the pandemic. Accordingly, the results should be interpreted with appropriate caution given the inherent limitations of the study design.

In summary, the LD dexamethasone regimen was non-inferior to the conventional HD regimen in preventing paclitaxel-induced HSRs and was associated with a lower incidence of steroid-related AEs. These findings support the use of reduced steroid dosing in routine clinical practice, particularly in patients at risk for steroid-related complications.

## Supplementary Information

Below is the link to the electronic supplementary material.

Supplementary file1 (DOCX 750 kb)

Supplementary file2 (DOCX 27.9 kb)

## Data Availability

Individual participant data are not permitted to be shared. Aggregated-level data can be requested from the corresponding authors.
