# Supplementary material for: Reduced-dose dexamethasone premedication for weekly paclitaxel: a retrospective cohort study of early hypersensitivity reactions and steroid-related toxicity
Source: Support Care Cancer. 2026 Jun 29;34(7):701. doi: 10.1007/s00520-026-10933-2 (PMC13315320; doi:10.1007/s00520-026-10933-2)
Supplement: Supplementary file 1 — Supplementary file1 (DOCX 750 kb) [file 520_2026_10933_MOESM1_ESM.docx]

**Journal name:** Supportive Care in Cancer

**Manuscript Title:** Noninferiority of reduced-dose dexamethasone premedication for weekly paclitaxel: Evaluating hypersensitivity reactions and steroid-related toxicity

**Authors:** Jeayoon Lee^1†^, Yijin An^1†^, In-Wha Kim^1^, Minoh Ko^1,2*^, Jung Mi Oh^1,3*^

^†^These authors have contributed equally to this work.

**Affiliations:**

^1^ College of Pharmacy and Research Institute of Pharmaceutical Sciences, Seoul National University, Seoul, Korea

^2^ College of Pharmacy, Daegu Catholic University, Gyeongsan, Republic of Korea

^3^ College of Pharmacy, Natural Products Research Institute, Seoul National University, Seoul, Republic of Korea.

**^*^Correspondence:** moko@cu.ac.kr (M.K.); jmoh@snu.ac.kr (J.M.O.)

**[Supplementary Figures Legends]**

**Supplementary Figure S1.** Directed Acyclic Graphs (DAGs) Illustrating the Causal Structure Between Dexamethasone Premedication Dose and Study Outcomes

**Supplementary Figure S2.** Covariate Balance Before and After IPTW

**Supplementary Figure S3.** ROC Curve for Propensity Score Model

**Supplementary Figure S4.** Time-Varying Hazard Ratios for Serious Bacterial Infections (High- vs. Low-Dose)

**Supplementary Figure S1. Directed Acyclic Graphs (DAGs) Illustrating the Causal Structure Between Dexamethasone Premedication Dose and Study Outcomes**

**Supplementary Figure S1A. DAG for Hypersensitivity Reaction**

**
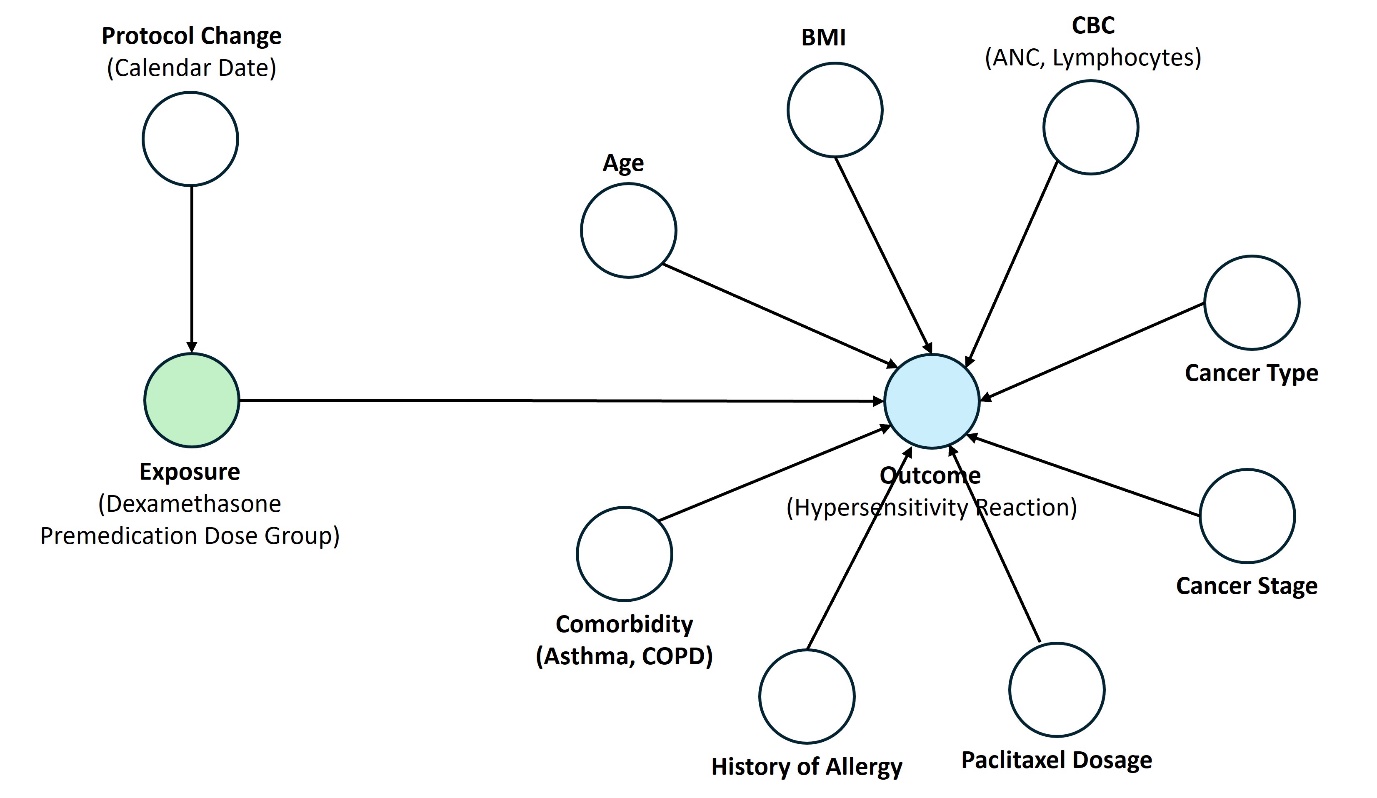
**

**Supplementary Figure S1B. DAG for Hyperglycemia**

**
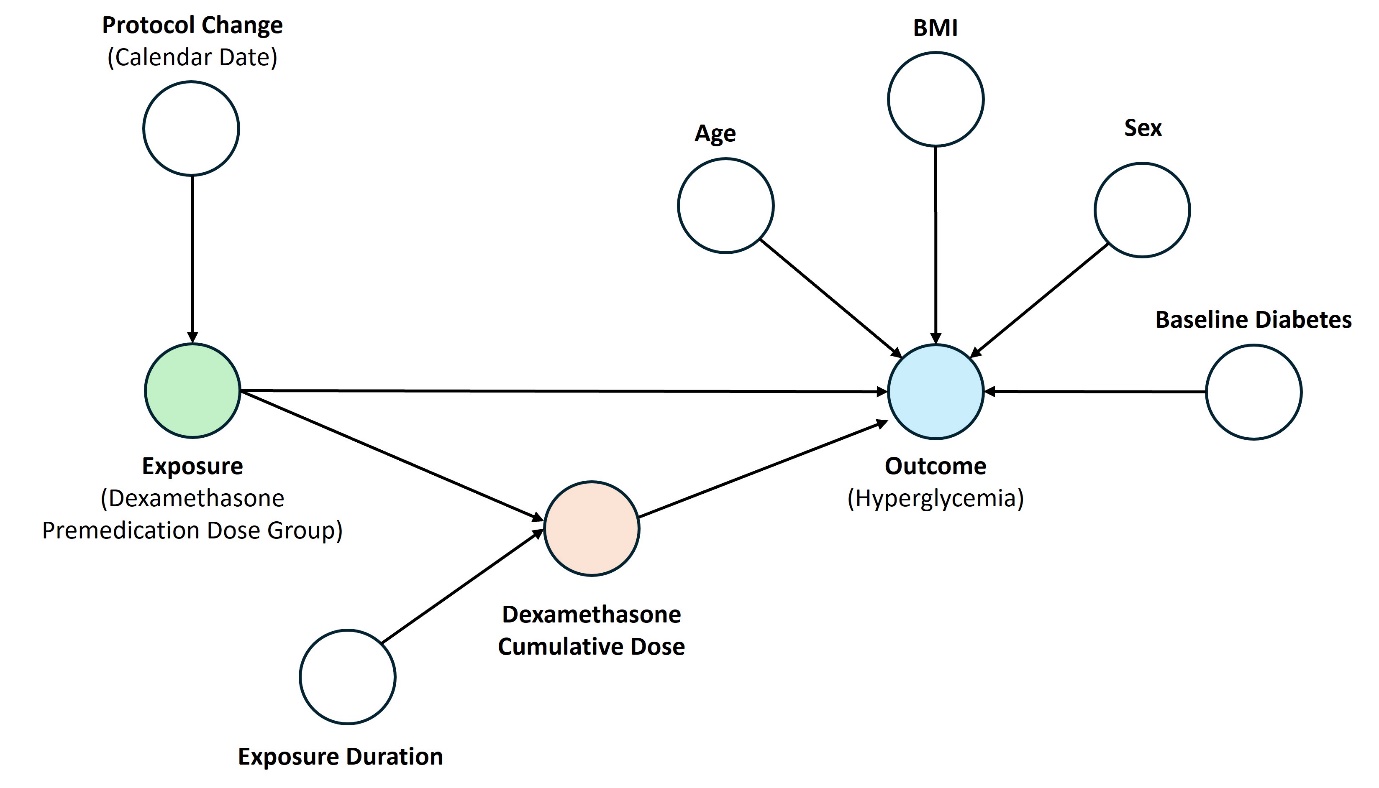
**

**Supplementary Figure S1C. DAG for Insomnia**

**
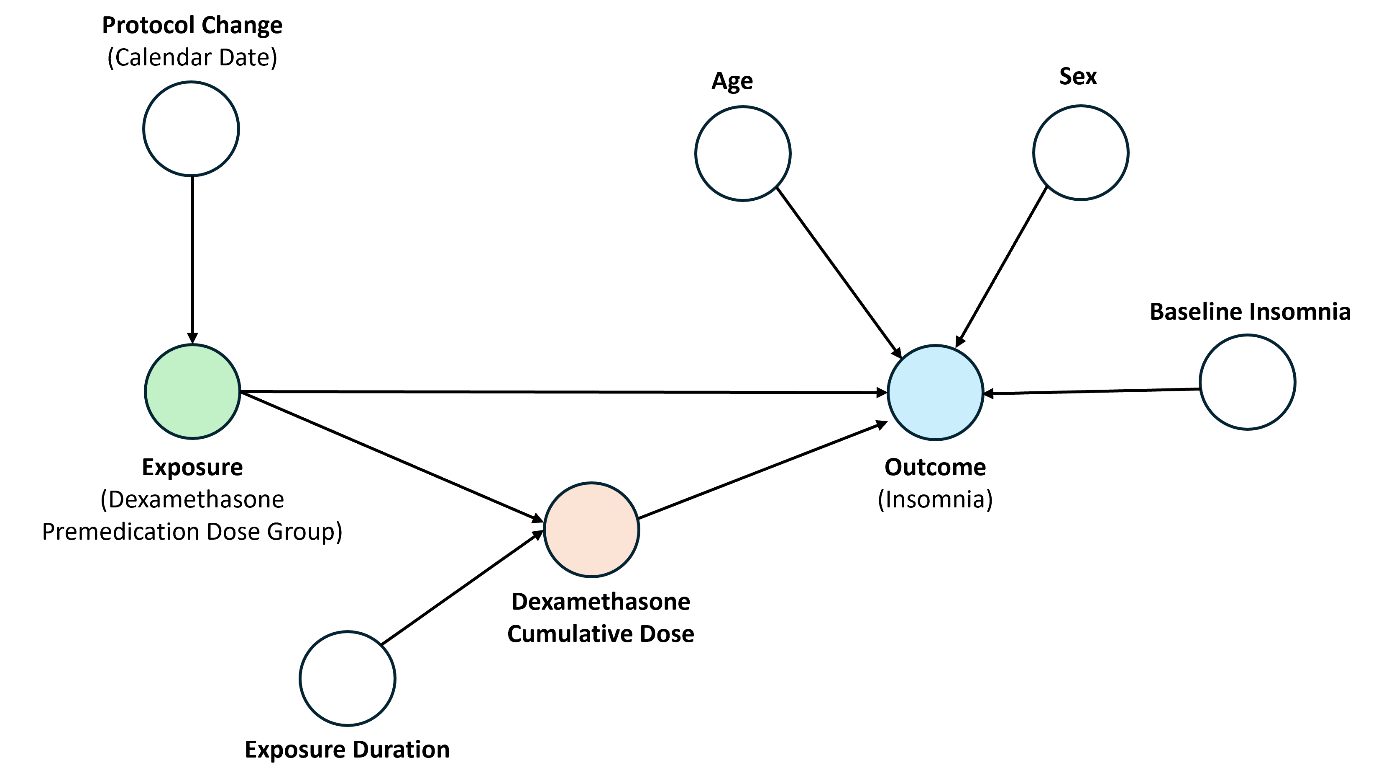
**

**Supplementary Figure S1D. DAG for Serious Infection**

**
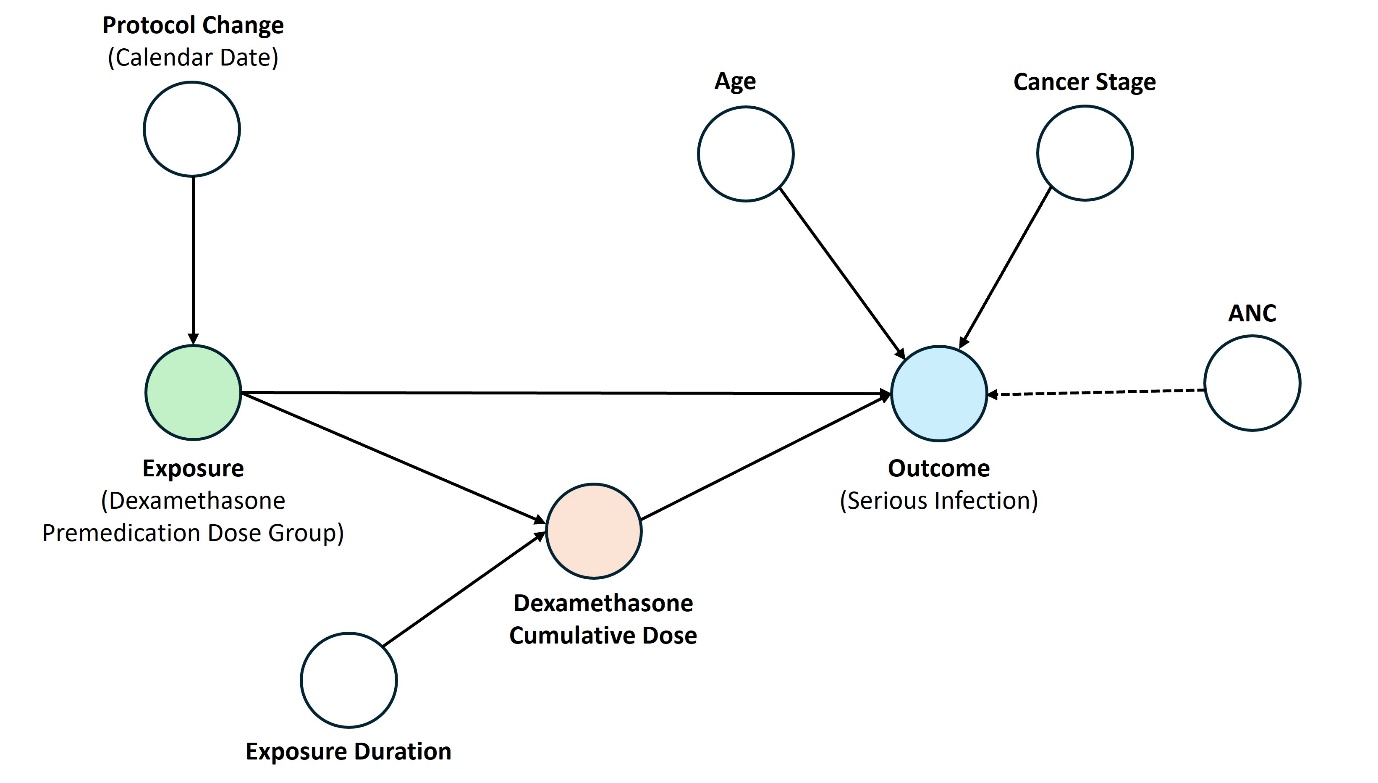
**

*Note: ANC was modeled as a time-varying covariate and thus represented with a dashed arrow to indicate its temporal dynamics.* Abbreviations: ANC, absolute neutrophil count

**Supplementary Figure S2. Covariate Balance Before and After IPTW**

Abbreviations: IPTW, inverse probability of treatment weighting

**
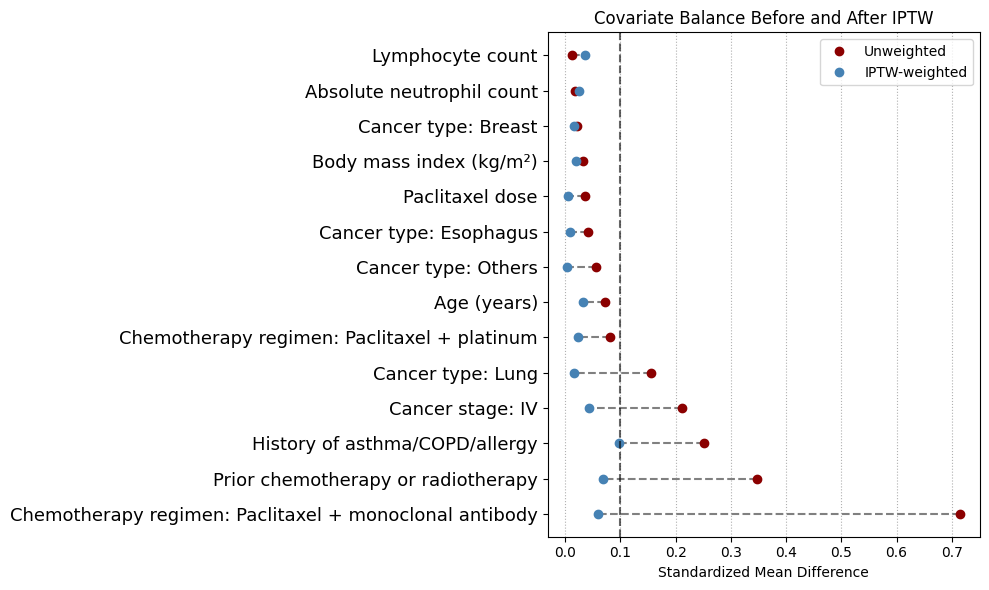
**

**Supplementary Figure S3. ROC Curve for Propensity Score Model.**

Abbreviations: ROC**,** receiver operating characteristic


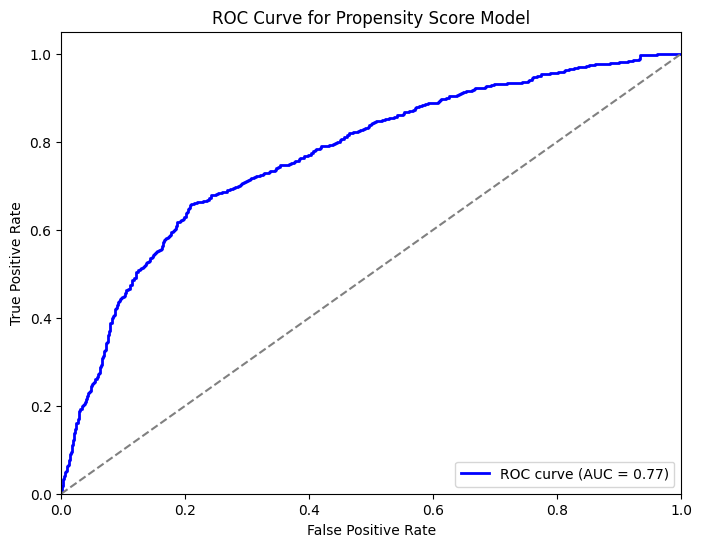


**Supplementary Figure S4. Time-Varying Hazard Ratios for Serious Bacterial Infection (High- vs. Low-Dose)**


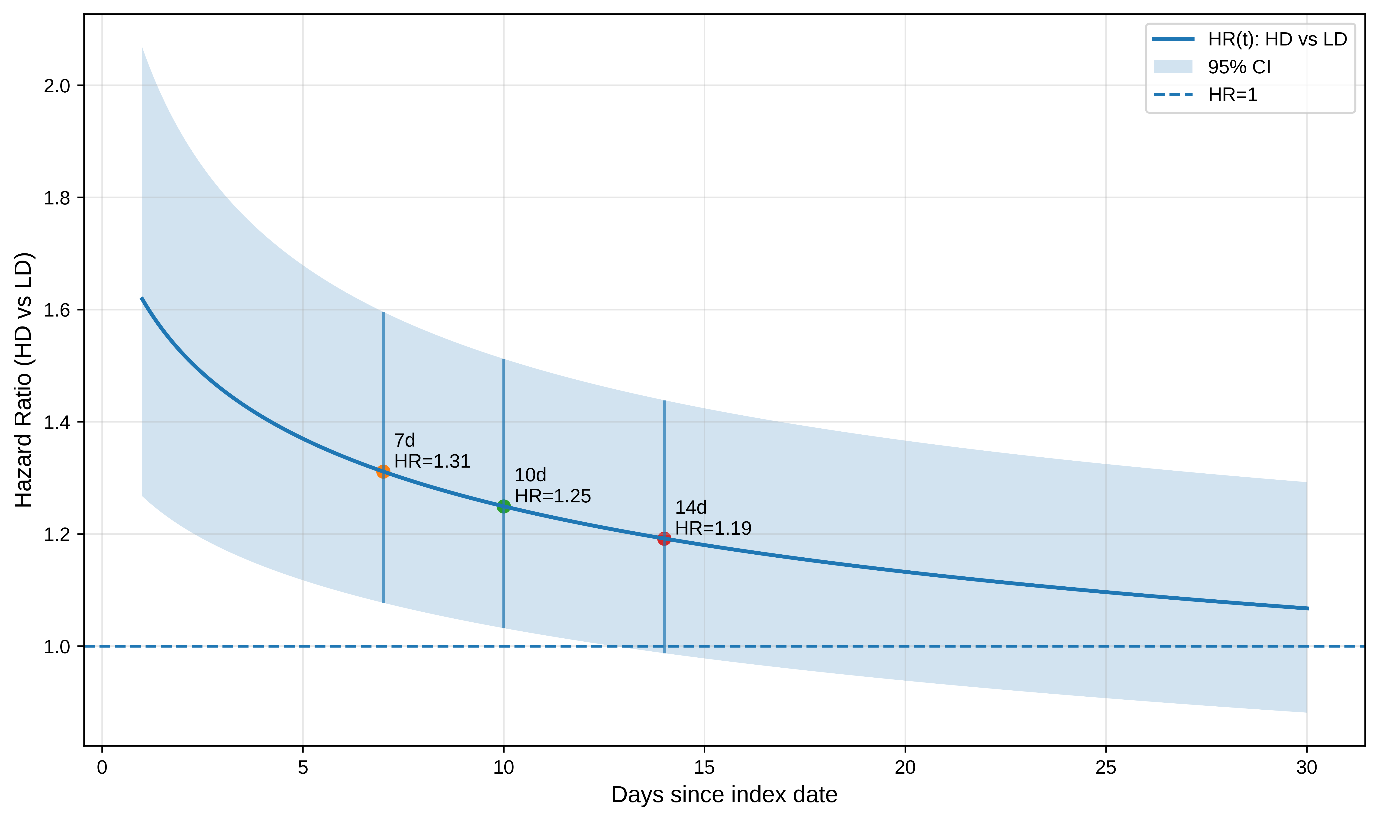


Time-varying hazard ratios (HRs) for serious bacterial infection comparing high-dose (HD) and low-dose (LD) dexamethasone groups across days since the index date. HRs were estimated using a Cox model incorporating a time-dependent interaction term. The dashed line represents an HR of 1.0, indicating no difference in risk between groups. HRs greater than 1.0 indicate a higher risk of bacterial infection in the HD group compared to the LD group.
