# Supplementary material for: Reduced-dose dexamethasone premedication for weekly paclitaxel: a retrospective cohort study of early hypersensitivity reactions and steroid-related toxicity
Source: Support Care Cancer. 2026 Jun 29;34(7):701. doi: 10.1007/s00520-026-10933-2 (PMC13315320; doi:10.1007/s00520-026-10933-2)
Supplement: Supplementary file 2 — Supplementary file2 (DOCX 27.9 kb) [file 520_2026_10933_MOESM2_ESM.docx]

**Journal name:** Supportive Care in Cancer

**Manuscript Title:** Noninferiority of reduced-dose dexamethasone premedication for weekly paclitaxel: Evaluating hypersensitivity reactions and steroid-related toxicity

**Authors:** Jeayoon Lee^1†^, Yijin An^1†^, In-Wha Kim^1^, Minoh Ko^1,2*^, Jung Mi Oh^1,3*^

^†^These authors have contributed equally to this work.

**Affiliations:**

^1^ College of Pharmacy and Research Institute of Pharmaceutical Sciences, Seoul National University, Seoul, Korea

^2^ College of Pharmacy, Daegu Catholic University, Gyeongsan, Republic of Korea

^3^ College of Pharmacy, Natural Products Research Institute, Seoul National University, Seoul, Republic of Korea.

**^*^Correspondence:** moko@cu.ac.kr (M.K.); jmoh@snu.ac.kr (J.M.O.)

**[Supplementary Tables Legends]**

**Supplementary Table S1.** Detailed Inclusion and Exclusion Criteria

**Supplementary Table S2.** Baseline Variable Definitions and Coding Criteria

**Supplementary Table S3.** Primary Outcome Definition and Grading Criteria (CTCAE v5.0)

**Supplementary Table S4.** Secondary Outcome Definitions

**Supplementary Materials**

**Supplementary Table S1. Detailed Inclusion and Exclusion Criteria**

| **Criteria Type** | **Detailed Criteria** |
| --- | --- |
| **Inclusion** | - Patients receiving weekly paclitaxel therapy at least twice at SNUH. - Treatment periods:   HD group: January 1, 2014, to May 31, 2018  LD group: January 1, 2020, to May 31, 2024. |
| **Exclusion** | - Prior paclitxel administration (including clinical trials) - Continuous oral dexamethasone (≥3 times). - Inaccessible EMR - Age <18 years at cohort entry. - Concurrent participation in other trials. - Severe HSR at first administration requiring desensitization at second administration. |

Abbreviations: HD, high-dose; LD, low-dose

**Supplementary Table S2. Baseline Variable Definitions and Coding Criteria**

| **Variable Category** | **Variable Name** | **Definition / Coding Criteria** |
| --- | --- | --- |
| **Demographics** | **Sex** | Male or Female |
|  | **Age** | Years at initiation of paclitaxel therapy |
|  | **Height** | Measured in centimeters (cm) |
|  | **Weight** | Measured in kilograms (kg) |
|  | **Body Mass Index** | Calculated as weight (kg) / height (m²) |
|  | **Body Surface Area** | Calculated using the Mosteller formula |
| **Chemotherapy-related** | **Cancer type** | Classified according to diagnostic codes (ICD-10) |
|  | **Chemotherapy regimen** | As recorded in EMR; reclassified for analysis |
|  | **Paclitaxel dose** | Dose per administration (mg/m²) |
|  | **Dexamethasone dose/duration** | Dose (mg) and number of administrations per cycle |
|  | **Surgery history** | Yes/No; any prior cancer-related surgery |
|  | **Radiotherapy history** | Yes/No; any prior cancer-related radiotherapy |
| **Comorbidities** | **Diabetes mellitus** | Fasting glucose >200 mg/dL and HbA1C ≥6.5%, or ICD-10 codes E11–E14 |
|  | **Hypertension** | ICD-10 code I10 |
|  | **Baseline insomnia** | Prescription of insomnia oral medication prior to paclitaxel initiation  Insomnia medication is as follows:  chloral syrup, flurazepam, triazolam, midazolam, zolpidem, zaleplon, eszopiclone, melatonin, clobazam, alprazolam, etizolam |
| **HSR-related factors** | **Allergy-related disease history** | ICD-10 codes J44–J46 (chronic airway disease/asthma) or L20 (atopic dermatitis) |
|  | **White blood cell count** | Measured in ×10³/μL, closest value prior to paclitaxel initiation |
|  | **Hemoglobin** | Measured in g/dL, closest value prior to paclitaxel initiation |
|  | **Absolute neutrophil count** | Measured in ×10³/μL, closest value prior to paclitaxel initiation |
|  | **Basophil count** | Measured in ×10³/μL, closest value prior to paclitaxel initiation |
|  | **Eosinophil count** | Measured in ×10³/μL, closest value prior to paclitaxel initiation |
|  | **Lymphocyte count** | Measured in ×10³/μL, closest value prior to paclitaxel initiation |

Abbreviations: EMR, electronic medical record; HSR, hypersensitivity reaction; ICD-10, International Classification of Diseases, 10th Revision.

**Supplementary Table S3.** **Primary Outcome Definition and Grading Criteria (CTCAE v5.0)**

**Symptom Categories and Examples:**

| **Category** | **Symptoms** |
| --- | --- |
| **Skin** | Pruritus, urticaria, rash |
| **Respiratory** | Dyspnea, bronchospasm |
| **Cardiovascular** | Hypotension/hypertension, tachycardia, chest pain, palpitations |
| **Systemic** | Flushing, fever, chills, weakness, syncope |
| **Gastrointestinal** | Nausea, vomiting, abdominal discomfort, diarrhea |
| **Neurological** | Extreme anxiety |

**HSR Grading:**

| **Grade** | **Description** |
| --- | --- |
| **1** | Mild symptoms; no intervention required |
| **2** | Moderate symptoms; non-invasive interventions required (infusion rate adjustment, oral medication) |
| **3** | Severe symptoms; invasive interventions or hospitalization required |

Abbreviations: HSR, hypersensitivity reaction

**Supplementary Table S4. Secondary Outcome Definitions**

| **Outcome** | **Definition and Criteria** |
| --- | --- |
| **Hyperglycemia** | Defined as ≥2 random glucose measurements >200 mg/dL within 30 days following the final administration of paclitaxel. The event date was defined as the date of the first glucose value exceeding 200 mg/dL. |
| **Serious Bacterial Infections** | Defined as the administration of at least two prescriptions of intravenous (IV) antimicrobial agents (Anatomical Therapeutic Chemical [ATC] codes: J01) within 7 days. The event date was defined as the earlier date between the two prescriptions. |
| ***Pneumocystis jirovecii pneumonia* (PJP) Infection** | Defined as the presence of at least one of the following within 30 days after paclitaxel administration:  (1) IV administration of sulfamethoxazole/trimethoprim at a therapeutic dose for the treatment of PJP.  (2) a diagnostic code indicating PJP.  (3) a positive result for *Pneumocystis jirovecii* by polymerase chain reaction (PCR) testing.  The event date was defined as the earliest date among the criteria met. |
| **Insomnia** | Defined as the presence of at least one prescription for an insomnia medication. Eligible medications were restricted to oral formulations listed under ATC codes N05B or N05C with an approved indication for insomnia. Medications prescribed solely for procedural or diagnostic purposes were excluded. The following agents were included: zolpidem, alprazolam, melatonin, and etizolam. |
